# FULL-THICKNESS ENDOSCOPIC GASTRIC RESECTION USING A STAPLER AND GASTROSTOMY: A FEASIBILITY STUDY

**DOI:** 10.1590/0102-672020180001e1386

**Published:** 2018-08-16

**Authors:** André M. WADA, Kiyoshi HASHIBA, Jose P. OTOCH, Horus BRASIL, Fernando P. MARSON, Jorge CASSAB, Ricardo ABDALLA, Everson L. A. ARTIFON

**Affiliations:** 1 Department of Surgery, University of São Paulo; 2 Sírio Libanês Institute for Teaching and Research, São Paulo, SP, Brazil.

**Keywords:** Obesity, Sleeve gastrectomy, Quality of life, Bariatric surgery, Obesidade, Gastrectomia vertical, Qualidade de vida, Cirurgia bariátrica

## Abstract

**Background:**

Laparoscopic sleeve gastrectomy (LSG) is currently the most frequently performed bariatric procedure in Turkey. The goal of weight reduction surgery is not only to decrease excess weight, but also to improve obesity related comorbidities and quality of life (QoL).

**Aim:**

To evaluate the impact of LSG on patient quality of life, weight loss, and comorbidities associated with morbid obesity according to the updated BAROS criteria.

**Methods:**

Eleven hundred thirty-eight adult patients were undergone to LSG by our bariatric surgery team between January 2013 and January 2016. A questionnaire (The Bariatric Analysis and Reporting Outcome System - BAROS) was published on social media. The data on postoperative complications were collected from hospital database.

**Results:**

Number of respondants was 562 (49.4%). Six of 1138 patients(0.5%) had leakage. All patients who had leakage were respondants. The overall complication rate was 7.7%. After a mean period of 7.4±5.3 months(1-30), mean excess weight loss was 71.3±27.1% (10.2-155.4). The respondants reported 772 comorbidities. Of these, 162 (30%) were improved, and 420 (54.4%) were resolved. The mean scores for QoL were significantly increased after LSG (range, p<0.05 to <0.001). Of the 562 patients, 26 (4.6%) were classified as failures; 86 (15.3%) fair; 196 (34.9%) good; 144 (25.6%) very good, and 110 (19.6%) excellent results according to the updated BAROS scoring system.

**Conclusion:**

LSG is a highly effective bariatric procedure in the manner of weight control, improvement in comorbidities and increasing of QoL in short- and mid-term.

## RESUMO

**Racional:**

A gastrectomia vertical laparoscópica (LSG) é atualmente o procedimento bariátrico mais frequentemente realizado na Turquia. O objetivo da operação de redução de peso não é apenas diminuir o excesso de peso, mas também melhorar as comorbidades e a qualidade de vida relacionadas à obesidade (QoL).

**Objetivo:**

Avaliar o impacto do LSG na qualidade de vida dos pacientes, perda de peso e comorbidades associadas à obesidade mórbida de acordo com os critérios BAROS atualizados.

**Métodos:**

Estudo não-randomizado de intervenção comportamental e de saúde pública. Um total de 1138 pacientes adultos foram submetidos a LSG entre janeiro de 2013 e janeiro de 2016. Um questionário (The Bariatric Analysis and Reporting Outcome System - BAROS foi utilizado. Os dados sobre complicações pós-operatórias foram coletados do banco de dados hospitalar.

**Resultados:**

Responderam ao questionário 562 (49,4%) pacientes. Seis de 1138 pacientes (0,5%) tiveram deiscência e todos estes responderam a pesquisa. A taxa geral de complicações foi de 7,7%. Após período médio de 7,4±5,3 meses (1-30), a perda média de excesso de peso foi de 71,3±27,1% (10,2-155,4). Os questionados relataram 772 comorbidades. Destes, 162 (30,0%) foram melhorados e 420 (54,4%) foram resolvidos.Os escores médios de QoL foram significativamente aumentados após LSG (p<0,05 a <0,001). Dentre os resultados dos 562 pacientes, 26 (4,6%) foram classificadas como falhas; 86 (15,3%) regular; 196 (34,9%) bom;144 (25,6%) muito bom; e 110 (19,6%) excelente de acordo com para o sistema de pontuação BAROS atualizado.

**Conclusão:**

O LSG é procedimento bariátrico altamente efetivo para controle de peso, melhora nas comorbidades e aumento da QoL em curto e meio prazos.

## INTRODUCTION

Gastric lesions such as gastrointestinal stromal tumors (GISTs) and other gastrointestinal tumors are treated via local endoscopic or surgical resection. One-layer^6, 16^ or full-thickness^4, 20,23,24,30^ resection can be performed in these cases. The standard treatments include long learning curves, time-consuming endoscopic procedures or invasive surgeries that may lead to complications. 

The aim of this study was to evaluate the feasibility and results of a full-thickness endoscopic gastric resection technique (FTEGR) using a stapler inserted through a gastrostomy. 

## METHODS

The protocol was approved by the Animal Care Institute Council of the Sírio Libanês Hospital, São Paulo, SP, Brazil. Ten domestic (Landrace) pigs weighing 35-40 kg were used in the study. All of the procedures were performed under general anesthesia and followed the same technique. Additionally, the antibiotic cephalosporin was administered in all animals. An oroesophageal overtube (Guardus; US Endoscopy, Mentor, OH, USA) was inserted under endoscopic guidance (GIF-150; Olympus, Tokyo, Japan).

### The FTEGR technique 

#### *Gastrostomy*

**FIGURE 1:**
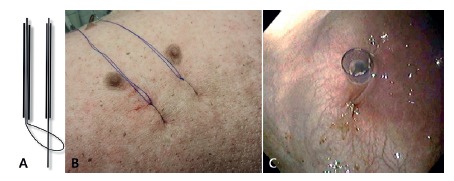
A) Two needles with 0 nylon threat for placing a “U” suture; B) outside view of the “U”sutures placed; C) inside view of the laparoscopic trocar inserted between the two “U”sutures through a gastrostomy

The first part of the procedure consists of a gastrostomy with transabdominal sutures^12^ ^,^ ^13^. A 27-gauge needle is inserted into the gastric lumen under endoscopic guidance in order to be a guide for the insertion of the other needles. A second 14-gauge needle with a 0 nylon thread is inserted 1.5 cm far or laterally from the previous needle. This endoscopic submucosal dissection needle has a suture loop in its interior, and it is inserted through the abdominal and gastric wall under endoscopic control. One additional 14-gauge needle with a 0 nylon thread in its inner channel is placed 1.5 cm from the previous location. The nylon suture of this needle is placed inside the previously inserted loop (Figure 1A) and pulled outside the skin to finish the “U” suture. A second “U” suture is placed in the same manner (Figure 1B). An incision made in the center of the area limited by the sutures is then used to insert a 12 mm laparoscopic trocar (Versaport Plus; Covidien, Miami, FL, USA) into the stomach lumen (Figure 1C). The sutures are completed by placing a manual knot in the abdominal and gastric walls. 

#### *T-tag stitch placement and traction of the resection area*

**FIGURE 2:**
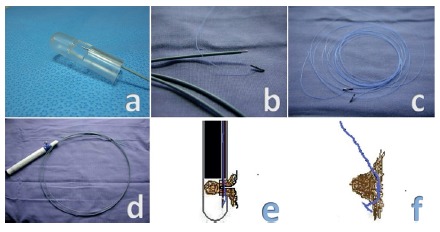
A) The plastic chamber; B) the metallic tube and the T-tag; C) the T-tag; D) the metallic tube; E) schematic figure showing the T-tag insertion in the gastric wall within the plastic chamber; F) T-tag inserted through gastric wall

Next, to pull the resection area, one or two sutures are placed on the stomach wall near the aimed resection area. For full-thickness sutures, a plastic chamber (prototype; Cook Medical Inc., Winston-Salem, NC, USA) (Figure 2A) is assembled at the distal tip of the endoscope. This chamber is 4.2 cm in length and has a side window measuring 10 mm×10 mm. The distance between the tip of the endoscope and proximal side of the window is 8 mm. The distance from the distal side to the tip of the chamber is 15 mm. Thus, a distal space is retained in the chamber to receive the needle inserted through the working channel of the chamber. This working channel is created in one side of the chamber wall where the wall is thickest. A T-tag is connected to a 2.0 nylon thread (Figure 2B) and is placed in a slot inside a 19-gauge metallic needle located within a plastic tube. This T-tag (Figure 2C) can be moved outside the metallic tube (Figure 2D) by pushing it through the suctioned gastric wall within the plastic chamber, and it remains in the distal space of the chamber (Figure 2E). The suction is released and the T-tag stitch is then pulled toward the animal’s mouth. By pulling this stitch (Figure 2F), the area forms a tent that includes all layers of the stomach at the highest point. 

#### *Resection and gastrostomy closure*

**FIGURE 3:**
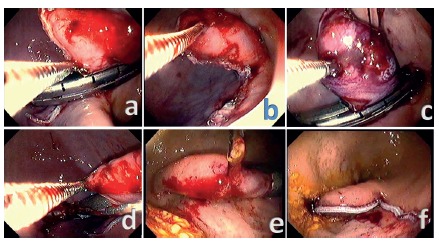
Full-thickness gastric resection using a stapler inserted through a gastrostomy. The linear stapler could resect all gastric layers by pulling the stitch towards the animal’s mouth, forming a tent, and the graspers help to accommodate the tissue (A, B, C and D). Specimen resected (E). The suture line achieved as a final result (F).

**FIGURE 4:**
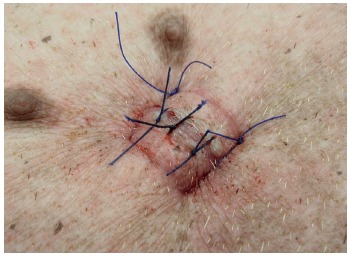
Gastrostomy’s closure using the previous 0 nylon thread “U” sutures and another two sutures between them just to close the skin

A 5 cm linear stapler (Endo Gia Universal; Covidien, Miami, FL, USA) is inserted through the gastrostomy trocar. It is placed parallel to the gastric wall surface around the tent base containing all gastric layers. The size of the tent is increased by pulling with a foreign grasper (FG-25C-1; Olympus, Tokyo, Japan) inserted through the working channel of the endoscope. The stapler is then used. If one magazine is not sufficient to complete the total resection of the specimen, another is used (Figure 3A, B, C, and D). Next, tissue is removed through the mouth by simply pulling the sutures (Figure 3E). The resected are is then examined (Figure 3F). The stapler is removed, followed by withdrawal of the trocar. The “U” nylon stitches at the gastrostomy are then untied and then square tied again but to close the gastrostomy site (Figure 4). On the day after the procedure, a regular diet is allowed, and parental analgesia is administered. The animals are sacrificed one month later after endoscopic control. 

## RESULTS

**FIGURE 5:**
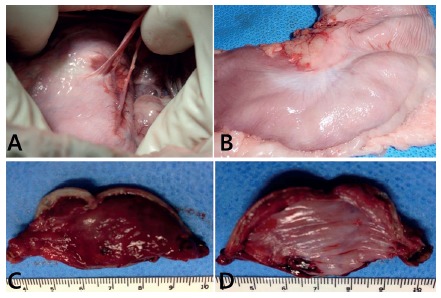
Specimen analysis: A) gastric walls after full-thickness resection with stapled sutures with omentum adhesions; B) without any adhesions C) full-thickness specimen of resected gastric wall viewed from interior (mucosal); D) exterior (serosal) view

FTEGR was accomplished in all animals, and all specimens included the serosa of the stomach (Figure 5). Immediate and complete closure of the resected area created by the stapler was observed in all animals.

The FTEGR specimens measured from 6×4.2 cm to 10×6.2 cm (Table 1). Calculations indicated that the average dimension of the specimens was 8 cm in length and 5 cm in width, with mean SD of 1.44 cm and 0.57 cm, respectively (Table 1). These mean (SD) are relatively small, indicating that specimen’s dimensions are distributed near average values, without a large dispersion (Table 1). The dotted lines in the Table 1 represent the trend lines for each variable, which, as this term implies, indicate the trends of growth in specimen sizes over the course of the study. An increasing trend in specimen length can be identified as the procedures progressed. However, for specimen width, the growth trend is much less pronounced; if we exclude sample 10, no clear trend is observed, with widths remaining nearly constant across specimens. 

**TABLE 1:** Specimen sizes and time of the procedure

Animal	Specimen length (cm)		Specimen width (cm)		Time of procedure (min)	
1	6.0		4.2		n/t	
2	6.0		4.5		n/t	
3	7.5		5.0		n/t	
4	7.0		5.5		n/t	
5	8.0		5.0		n/t	
6	8.2		5.0		n/t	
7	8.5		5.2		81	
8	10.0		5.2		85	
9	9.0		4.5		72	
10	10.0		6.2		75	
		Specimen length (cm)		Specimen width (cm)		Time of procedure(min)
Average		8.0		5.0		78.25
Standard Deviation		1.44		0.57		5.85
Minimum		6.0		4.2		72
Maximum		10.0		6.2		85
						

N/t: not timed

Time was spent in preparing the procedure due to the use of these prototype devices and for this reason only in the last four procedures it was measured (Table 1). The mean time to perform FTEGR was 78 ± 5.85 min (Table 1, 72-85 min). Only in the first procedure a self-limited bleeding was observed in the staple line. No other adverse events were observed. Additionally, a scar was observed at the resection site during the day 30 follow-up endoscopy and laparotomy. At sacrifice, there were no small bowel adhesions in the resection line; only a small number of omentum adhesions were eventually found in the resection line (Figure 5). 

## DISCUSSION

Less-invasive procedures should be used whenever possible. This approach has the potential to decrease risks, shorten hospital stays, and reduce costs. Endoscopic approach is usually less invasive than surgery, even when a laparoscopic procedure is required. 

An important advantage of FTEGR is the immediate closure of the resected area preventing leakage and peritoneal cavity contamination. On the other hand, as the resected specimen is retrieved trough the mouth no cancer cell may spill into the peritoneal cavity, preventing cancer dissemination. This is a concern since reports of peritoneal seeding has been reported after percutaneous diagnostic FNA biopsy^8^ and port-site metastasis after laparoscopic surgery for gastrointestinal malignancy^7^.

In selected cases of high grade dysplasia or non-invasive adenocarcinoma of the stomach, endoscopic treatment can be performed through endoscopic mucosal resection (EMR) or endoscopic submucosal dissection (ESD)^10^ ^,^ ^27^, both of which are popular procedures, worldwide. Despite representing a breakthrough in the field of endoscopy, ESD may be a time-consuming procedure, depending on the location of the lesion, its size, submucosal fibrosis and submucosal invasive cancer requiring expertise and may lead to significant complications such as perforation and bleeding^17^ ^,^ ^18^ ^,^ ^29^. Thus, ESD is not adopted by all endoscopic centers at which EMR is routinely performed. 

The gold standard for the treatment of gastric lesions such as GISTs larger than 2 cm is surgical resection according to current ESMO and NCCA guidelines^9, 22^. However, when GIST is histologically proven the European and Japanese guidelines recommend resection regardless of its size. Lately, several endoscopic and combined endoscopic and laparoscopic resections techniques have been proposed^2^ ^,^ ^11^ ^,^ ^15^ ^,^ ^19^ ^,^ ^21^ ^,^ ^24^ ^,^ ^26^ ^,^ ^29^ ^,^ ^31^ ^,^ ^32^. However, these types of treatments exhibit some complications^1^ ^,^ ^14^.

Gastric lesions located at the greater curvature, and most parts of the anterior e and posterior body appear to be suitable for treatment using the FTEGR. 

The position of the lesion to be resected is a limitation because the traction of the target area to the gastric lumen central axis can be challenging. In addition, T-tag deployment is difficult when the endoscope is flexed. The stapler has limited maneuverability despite some angulation allowed by its body. 

In fact, there must be some places that cannot be reached with the devices used in this study, as the fundus, the cardia, the lesser curvature and near the pylorus. However it is important to emphasize that the management of these areas depends also on the possibility of placing a suture in these areas and the way of making the traction and the position of the patient. In the study a standardized procedure was followed. However, for specific places, the technique could be changed. Thus, the wall traction could be made directly by the endoscope, using a foreign grasper or a snare through the working channel. Moreover, the suture for traction could be placed by a suturing device, such as Endo Stitch (Endo Stitch 10 mm Suturing Device, Covidien, Miami, FL, USA), inserted through the trocar. 

Recently, endoscopic treatment for small gastric sub epithelial tumors was proposed^5^. Using this technique, the lesion is aspirated inside a cap, and a loop is tightened at the base, followed by an incision of the overlying mucosa using a needle knife to perform biopsies or enucleation of the lesion. 

FTEGR could be used for the treatment of early gastric cancer and sub-epithelial lesions in selected cases and for the obesity treatment. The resection of a longitudinal strip in the gastric body, including the greater curvature, part of the anterior and part of the posterior wall provides lumen restriction and decrease stomach capacity. The process can result in a tunnel shaped gastric body similar to the endoscopic sleeve gastroplasty procedure^3^. However, further refinement of the T-tag placement and improved stapler flexibility are necessary to allow resection of larger areas and of all stomach places.

This FTEGR study demonstrates that the use of a stapler inserted while performing a gastrostomy can be combined with the wall traction to resect a large, full-layer gastric specimen. Larger specimens can be resected if more stitches are placed for tissue tenting. This experimental technique does not require meticulous dissection and may be associated with fewer complications than ESD. Additionally, the learning curve for the procedure seems to be short, which may allow it to be performed by more endoscopists. Moreover, the FTGER seems to be less invasive than laparoscopic surgery and may allow faster recovery.

More studies are needed to confirm these data. 

## CONCLUSION

FTEGR is a feasible technique for the resection of full-layer gastric specimens and appears to be safe. It can be an alternative to endoscopic submucosal dissection and surgical full-thickness gastric partial resection in selected cases.
